# Rule-out and rule-in of carotid near-occlusion using color duplex ultrasound

**DOI:** 10.1007/s00234-025-03612-2

**Published:** 2025-04-16

**Authors:** Johan Skoog, Davide Vanoli, Alexander Henze, Allan J. Fox, Elias Johansson

**Affiliations:** 1 Department of Clinical Physiology, Sahlgrenska University Hospital, Region Västra Götaland, Gothenburg, Sweden; 2 Department of Molecular and Clinical Medicine, Institute of Medicine, Sahlgrenska Academy, University of Gothenburg, Gothenburg, Sweden; 3 Department of Public Health and Clinical Medicine, Umeå University, Umeå, Sweden; 4 Institution of Radiation Sciences, Department of Diagnostic and Intervention, Umeå University, Umeå, Sweden; 5 Sunnybrook Health Science Center, Department of Medical Imaging, University of Toronto, Toronto, ON Canada; 6 Institution of Clinical Science, Department of Neurosciences, Umeå University, Umeå, Sweden; 7 Wallenberg Center of Molecular Medicine, Umeå University, Umeå, Sweden; 8 Institute of Neuroscience and Physiology, Department of Clinical Neuroscience, Sahlgrenska Academy, Gothenburg, Sweden; 9 Department of Clinical Physiology, Sahlgrenska University Hospital, Blå stråket 5, Gothenburg, 413 45 Sweden

**Keywords:** Carotid near-occlusion, Colour duplex ultrasound, CT angiography, Carotid stenosis, Stroke

## Abstract

**Purpose:**

Diagnosing carotid near-occlusion (CNO) with colour duplex ultrasound (CDU) is challenging. We hypothesised that CNO is associated with a reduced distal internal carotid artery (ICA) velocity and aimed to assess if distal velocity is able to diagnose CNO accurately. If not, we aimed to develop CDU rule-out and rule-in criteria to diagnose CNO.

**Methods:**

This is a prospective cross-sectional study in consecutive participants with suspected ≥ 50% carotid stenosis on CT angiography (CTA). CDU velocities in the common carotid artery, the stenosis and distal to the stenosis were examined. CTAs were assessed for CNO, serving as a reference test. If no CDU parameter was both sensitive and specific for CNO, rule-out (98% sensitive) and rule-in (99% specific) criteria were created.

**Results:**

Of the 315 included participants with ≥ 50% stenosis, 190 (60%) were conventional ≥ 50% stenosis and 125 (40%) CNO. No CDU parameter was both sensitive and specific for CNO. The best exclusion criteria were stenosis end diastolic velocity (EDV) ≤ 63 cm/s and/or distal peak systolic velocity (PSV) > 23 cm/s, seen in 115 (38%) participants. The best rule-in criteria were stenosis EDV ≥ 280 cm/s and/or distal PSV ≤ 23 cm/s, seen in 35 (12%) participants. Of the remaining participants, 143 (47%) were uncertain (74 CNOs) and 9 (3%) were misdiagnosed as carotid occlusion (all CNOs).

**Conclusions:**

CDU alone cannot diagnose CNO but can rule in or rule out CNO in half of participants with ≥ 50% stenosis. These criteria are intended for inclusion in an algorithm, sorting cases needing further exams, such as CTA and/or phase-contrast magnetic resonance angiography.

**Supplementary Information:**

The online version contains supplementary material available at 10.1007/s00234-025-03612-2.

## Introduction

Carotid near-occlusion (CNO) is a severe carotid stenosis in which the ICA distal to the stenosis is reduced in size (collapsed) [1–3]. In contrast, conventional ≥ 50% stenosis is not characterized by a collapsed vessel distal to the stenosis [1–3]. CNO can further be classified as with or without full collapse [4], where the reduction in ICA caliber is severe in the former and more moderate in the latter [5]. Differentiating CNO from conventional stenosis is important due to differences in treatment recommendations: Conservative treatment is generally recommended for symptomatic CNOs, while carotid endarterectomy/carotid artery stenting are recommended for symptomatic conventional ≥ 50% stenosis [6].

Although CNO have been presented as relatively rare, recent findings suggest a prevalence of around 30% among participants with symptomatic ≥ 50% stenosis [4]. Angiography, such as CTA, can diagnose CNO, but is often claimed to be difficult to apply [7]. Recently, phase contrast magnetic resonance angiography (PC-MRA) has been suggested as an accurate (> 95%) method to diagnose CNO [8]. Nevertheless, color duplex ultrasound (CDU) is often the first-line imaging modality in patients with suspected carotid artery disease [6, 9]. CDU findings of a severe stenosis with low stenosis flow velocity are very specific for CNOs, but are foremost seen in CNOs with full collapse [10, 11]. Most CNOs have a peak systolic velocity (PSV) ≥ 125 cm/s, just as conventional ≥ 50% stenosis [12]. No traditional velocity parameter originating from the stenosis or common carotid artery (CCA) have rendered a sensitivity and specificity above 75% [5]. However, a small proof-of-concept study has shown the velocity distal to the stenosis to be reduced in CNO compared to conventional ≥ 50% stenosis [13]. This has not yet been assessed in a larger cohort.

In addition, given the high accuracy of PC-MRA for CNO, it might become the future method of choice for CNO diagnostics. If CDU is not similarly accurate, it seems reasonable to concede that CDU is not alone sufficient to diagnose CNO but should rather serve as a selection tool in a diagnostic algorithm. To do so, diagnostic criteria aimed to rule out and rule-in CNO are required, but no such criteria exist.

We hypothesised that CNO is associated with a CDU reduced distal ICA velocity compared to conventional ≥ 50% stenosis, and that this correlation is sufficiently strong for single thresholds to be both sensitive and specific for CNO. If not, the study also aimed to develop rule-out and rule-in criteria to diagnose CNO.

## Materials and methods

### Study design and population

Single-centre cross-sectional study. We prospectively included consecutive patients with ≥ 50% carotid stenosis between 2018 and 2022 at University Hospital of Northern Sweden, Umeå, Sweden. Study logistics was handled at the stroke unit, a tertiary stroke unit and the sole provider of preoperative carotid evaluations for 11 referring hospitals (population 880 000). Most participants had symptomatic stenosis and were assessed for carotid surgery. Some participants turned out to have asymptomatic stenosis during this preoperative assessment, and some participants with known asymptomatic stenosis were summoned for study purposes. Inclusion criteria for being asked to participate was 50–100% carotid stenosis on at least one side on any exam type. Of participants who provided informed consent, we excluded those that did not undergo CTA and CDU, those without a ≥ 50% atherosclerotic stenosis on at least one side or unclear cause of small distal ICA on the study-assessments of CTA. Both CDU and CTA were part of the clinical routine but was arranged as part of the study when this routine was not well adhered to.

This was one of several preplanned analyses of the same material. The sample size was calculated based on all preplanned analyses and set to accommodate the analysis requiring the most participants, which was not this analysis, which is why this study was overpowered. The regional ethics board in Umeå, Sweden approved the study.

### CDU

CDU examinations were performed by several vascular technologists in the Department of Clinical Physiology using a LOGIQ E9 and E10 ultrasound system (GE Healthcare, Wauwatosa, Wisconsin). All examiners were blinded to the study CTA assessments. Ultrasound measurements were conducted by standard recommendations, utilizing grayscale, colour Doppler, and spectral Doppler imaging [14]. Additional flow velocity measurements were obtained in the distal ICA. Distal ICA was measured as far distally as possible but without sacrificing image quality. PSV and end diastolic velocity (EDV) for these measurements were prospectively entered in a study database, usually just after the exam. When this was overlooked or to clarify details, a subset (approximately 20%) was re-reviewed. Carotid occlusion was diagnosed if no measurable flow velocity was detected in or distal to the stenosis. Here, high-sensitive machine settings and ultrasound contrast were used to detect minute flow.

### CTA

CTA was performed at several radiology departments at the same or referring hospitals and hence were done with various machines and clinical protocol variants. All images were assessed for study purposes by three observers, blinded to each other and CDU findings. EJ (10 years’ experience) assessed all exams, AJF (> 40 years’ experience) all with possible CNO and some controls during the first half of the study and AH (5 years’ experience) all exams during the second half of the study.

CNO was diagnosed when there was a severe stenosis associated with a reduced ICA diameter beyond the stenosis, in the absence of other features to suggest alternative causes (dissection, anatomical variations or others), presented in detail elsewhere (Supplemental Fig. 1A-B) [8]. In short, the whole exam was assessed to determine if the distal ICA was small and if the severe proximal atherosclerotic stenosis was the most reasonable cause. Stenosis severity, distal ICA diameter, ICA ratio (side-to-side) and ECA ratio (ICA-ECA) were most considered, and additionally, the Circle of Willis and possible distal ICA disease. The most common differential diagnoses for small distal ICA were dissection and anatomical variation, i.e., asymmetric Circle of Willis (Supplemental Fig. 1C-D). Anatomical variations with smaller ICA with stenosis were then not CNO but considered as conventional stenosis [15].

A 2-sided conservative approach was used: Both conventional stenosis and CNO were diagnosed only when features were sufficiently clear. The non-diagnosis of unclear cause of small distal ICA was set in two situations: First, when distal ICA was small, but the reason was unclear (divergent features). Second, cases where the distal ICA varied in diameter, and it was unclear if it should be considered small (but would have been a CNO if it was considered small). Cases with unclear causes of small distal ICA were excluded (Supplemental Fig. 1E-F). This conservative approach appears reasonable, as previous studies analysing postoperative CTA have reported a roughly equal distribution of conventional stenosis and CNO among participants with unclear cause of small distal ICA on preoperative CTA [8]. Among CNO, full collapse was diagnosed according to the 2022 Johansson definition [16]. Occlusion was diagnosed when no contrast was visible beyond the stenosis, usually confirmed with serial imaging CTA (a study exam) and occasionally with conventional angiography. Artery measurements were captured by EJ. Tiny artery segments that where the lumen had lower opacification than surrounding arteries (presumed partial volume effect) were assigned 0.5 mm in diameter when visible and 0.2 mm when not visible (but obviously existent from context) [8].

The side with the most severe stenosis (excluding occlusion) on CTA was chosen as index side. If the most severe stenosis was unclear cause of small distal ICA, the contralateral side was occasionally used for comparison (if it had a conventional > 50% stenosis).

### Statistical analyses

Most CDU variables and all laboratory data were non-normally distributed and presented as median (interquartile range (IQR)). Categorical data were expressed as numbers and percentages. Differences between groups were assessed with Mann-Whitney *U* test and Fisher’s exact test, as appropriate.

Diagnostic analyses were done in three steps. First, receiver operating characteristic curves (ROC) and estimated area under the curve (AUC) were used to evaluate the diagnostic performance of single CDU variables. Thresholds were set for maximum Youden index and resulting sensitivity, specificity, positive (PPV) and negative predictive values (NPV) with corresponding 95% CI were calculated. Second, the CDU variable with highest AUC was used to create one rule-in criterion (≥ 99% specificity) and one rule-out criterion (≥ 98% sensitivity). Cases between these criteria were considered unclear. Third, step 2 was repeated for the combinations of all CDU variables with an AUC > 0.7 where the thresholds of both parameters were adjusted to create the best combination, defined as the smallest number of unclear cases still achieving ≥ 99% specificity and ≥ 98% sensitivity.

A *p* value < 0.05 was considered statistically significant. Statistical analyses were carried out using the SPSS 27.0 for Windows software (IBM Corp., Armonk, NY, USA). The threshold adjustment for two parameters in combination was performed with a dedicated Excel-based software (Microsoft Corp., Redmond, WA, USA).

## Results

**Fig. 1:**
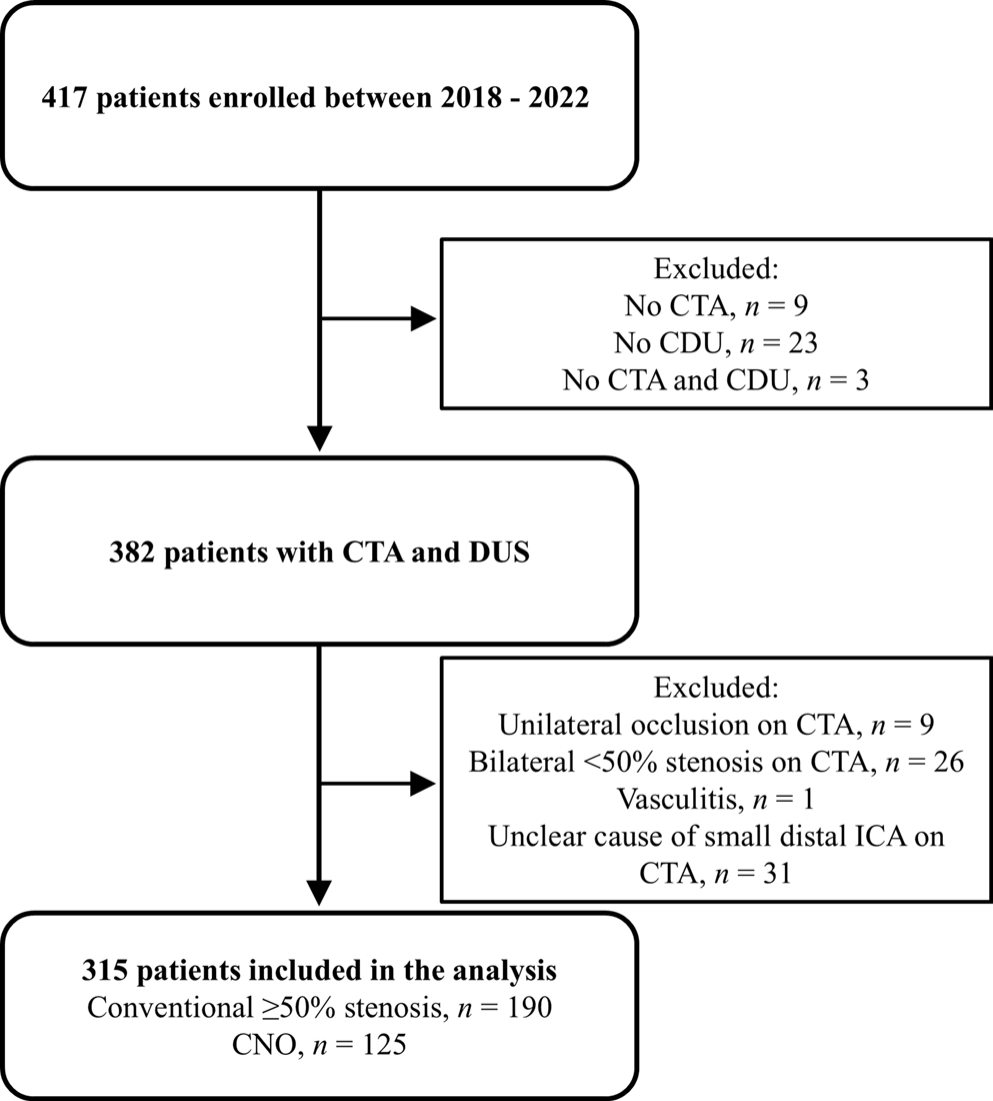
Study flow chart with the number of included and excluded participants and final adjudicated diagnosis. CDU, color duplex ultrasound; CNO, carotid near-occlusion; CTA, CT angiography

Of the 417 consecutive participants enrolled in the study with ≥ 50% stenosis on at least one side on any exam, 315 were included in the analysis (Fig. 1). The final adjudicated diagnosis, based on CTA, was conventional ≥ 50% stenosis in 190 participants (60%) and CNO in 125 (40%). Unilateral ≥ 50% stenosis or occlusion were observed in 222 of these participants. Except for CTA measurements, there were no differences in baseline characteristics between the stenosis groups (Table 1). Nine participants with CNO on CTA were misclassified as occlusions during CDU. The Kappa value for interrater reliability in diagnosing CNO on CTA was 0.70 (95% CI 0.63–0.77) between observer 1 and observer 2 and 3.

All CDU parameters differed significantly between participants with CNO and conventional ≥ 50% stenosis (all *p* <.001, Table 2). Compared with ≥ 50% conventional stenosis, participants with CNO had higher stenosis velocities and stenosis-CCA ratios, lower CCA and distal ICA velocities, lower distal PI and lower distal-stenosis ratios (Table 2 and Supplemental Fig. 2).

**Fig. 2:**
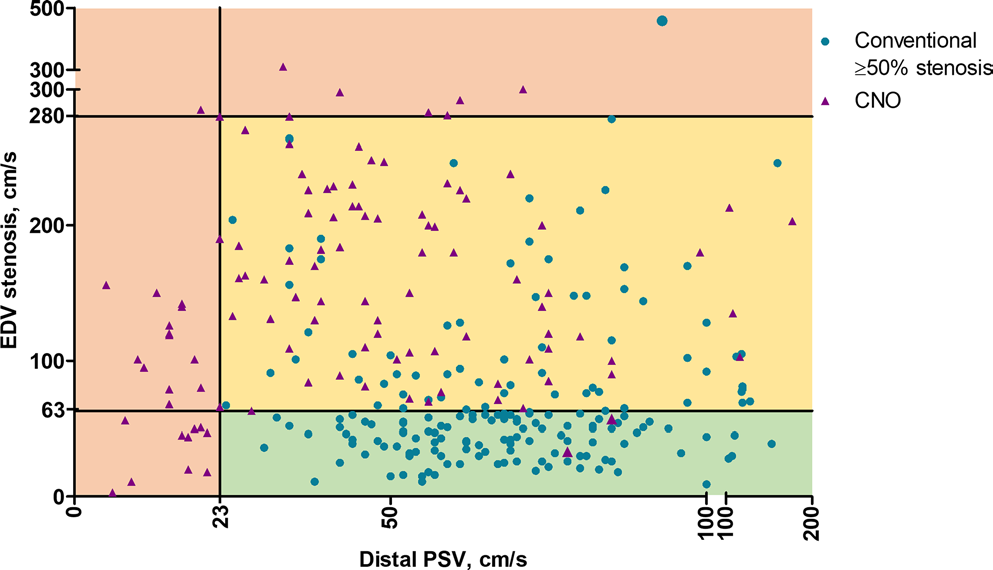
Distribution of participants according to the final CDU algorithm. Rule-out (stenosis EDV ≤ 63 cm/s and distal PSV > 23 cm/s, green). Rule-in (stenosis EDV ≥ 280 cm/s and/or distal PSV ≤ 23 cm/s, red). Uncertain (yellow). CNO, carotid near-occlusion; EDV, end-diastolic velocity; PSV, peak systolic velocity

**Fig. 3:**
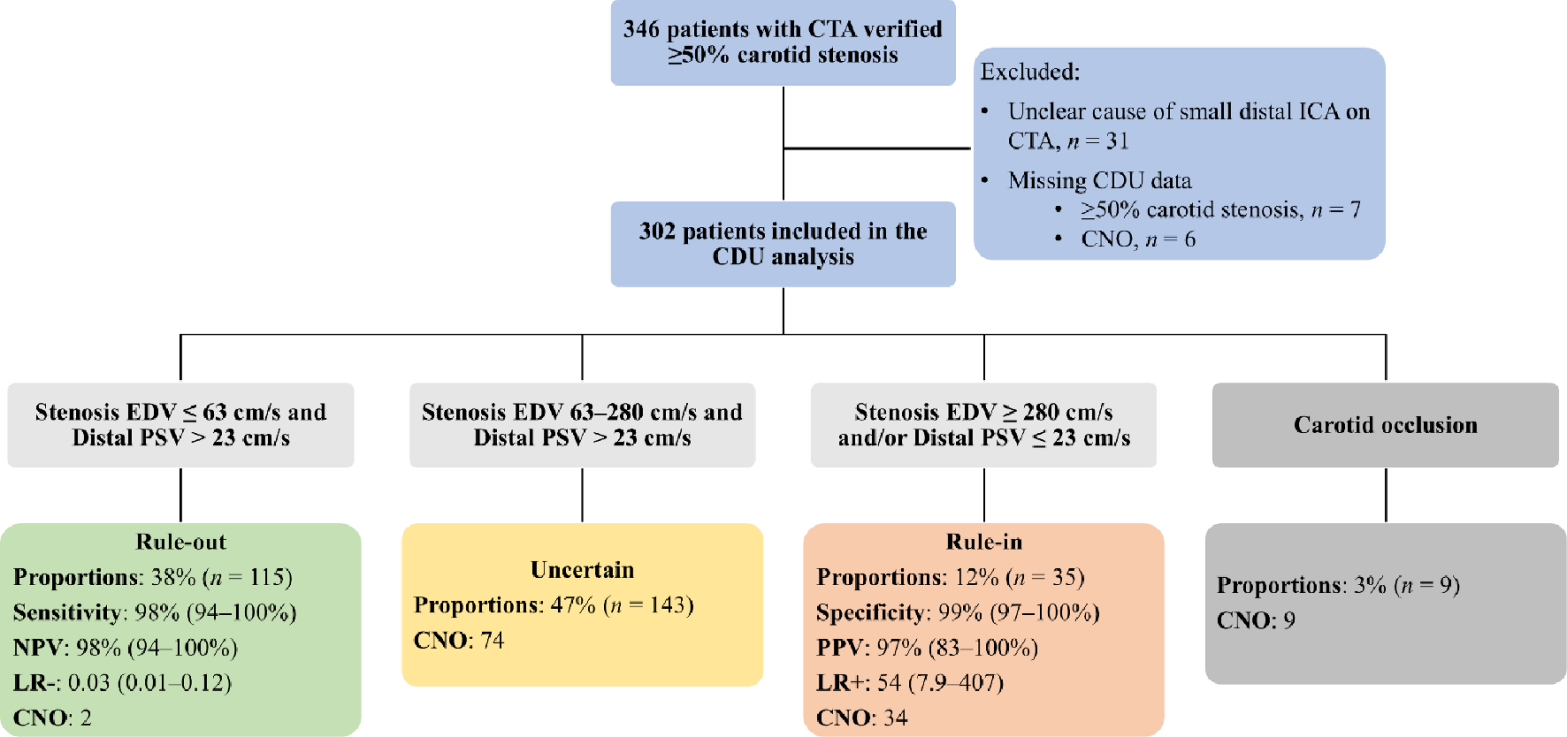
Algorithm for diagnosis of CNO using CDU in participants with CTA confirmed ≥ 50% stenosis. CDU, colour duplex ultrasound; CNO, carotid near-occlusion; CTA, CT angiography; EDV, end-diastolic velocity; LR+, positive likelihood ratio; LR-, negative likelihood ratio; NPV, negative predictive value; PPV, positive predictive value; PSV, peak systolic velocity

**Fig. 4:**
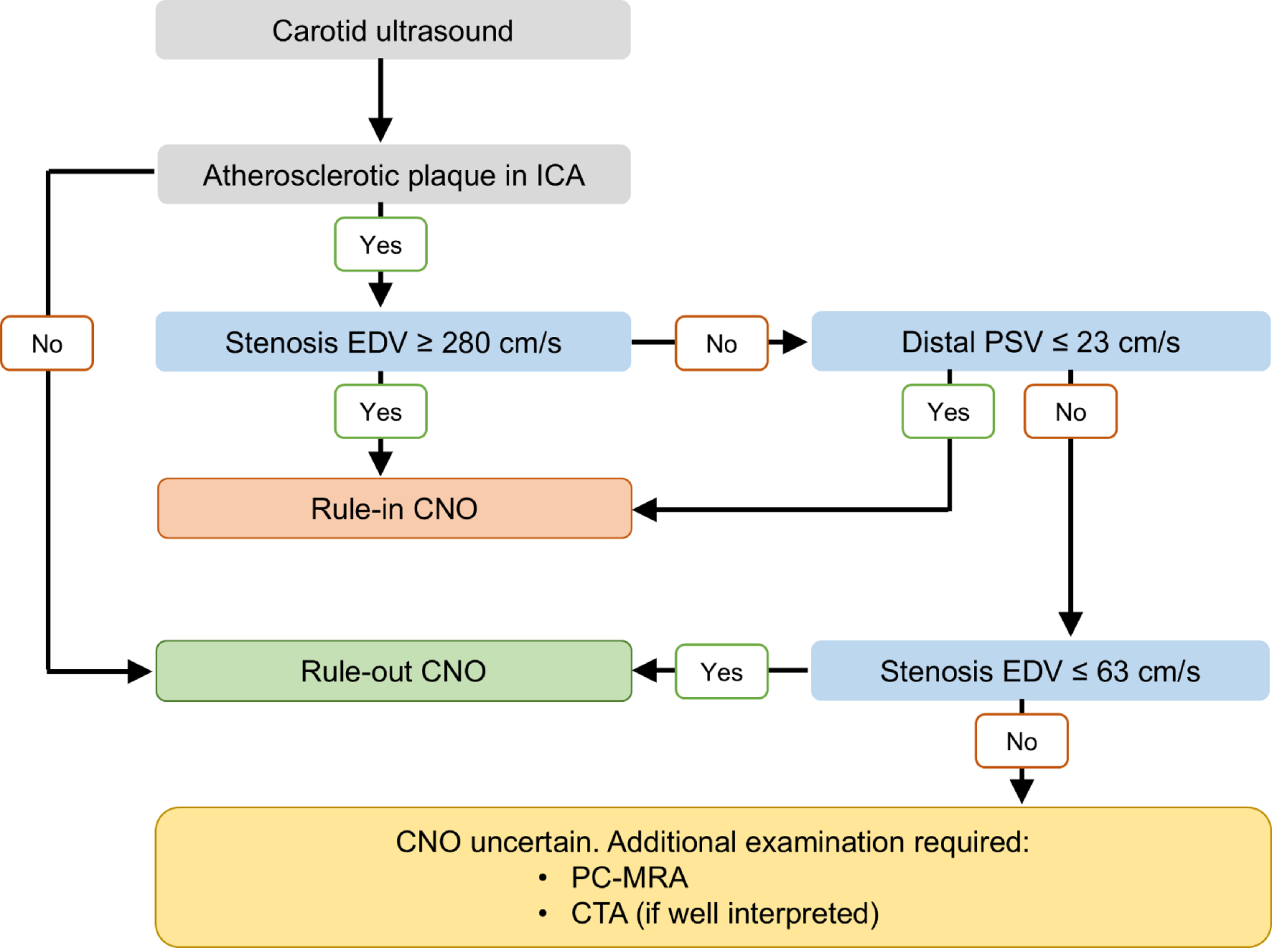
Flowchart illustrating the proposed diagnostic algorithm for carotid near occlusion using CDU. CDU, colour duplex ultrasound; CTA, CT angiography; EDV, end-diastolic velocity; PC-MRA, phase contrast magnetic resonance angiography; PSV, peak systolic velocity

The diagnostic performance of each CDU parameter to diagnose CNO was low-to-moderate, with several AUCs ranging between 0.80 and 0.89 (Table 3 and Supplemental Fig. 3). No differences in AUC were observed in the subgroup analysis stratified by symptoms related to the investigated side of the ICA (Supplemental Table 1). With thresholds set for highest Youden index, the best parameter (distal PSV ratio ≤ 0.17) was 77% sensitive and 89% specific for CNO. With thresholds set for rule-in criterion (≤ 0.062 for ≥ 99% specificity) and rule-out criterion (≥ 0.42 for ≥ 98% sensitivity), distal PSV ratio could rule-out 39% of cases, rule-in 8% and 53% were uncertain (Criteria 1, Table 4). The number of uncertain cases was somewhat lower when combining distal PSV with either stenosis EDV (48% uncertain) or EDV ratio (51% uncertain; criteria 2–3, Table 4). Since distal PSV for rule-in and rule-out was very similar in criteria 2, we created an easy-to-use variant where distal PSV was the same (≥ 23 cm/s) and slightly changed rule-out stenosis EDV. This resulted in the same accuracy but marginally higher uncertain cases (49%, criteria 4, Table 4 and Fig. 2). We call this the “best criteria”. The practical outcome of the best criteria, including that some CNOs were mistaken for occlusion, is summarized in Fig. 3, and a flowchart depicting the proposed diagnostic algorithm is presented in Fig. 4. In addition, five other combinations had ≤ 58% uncertain, i.e., < 10% worse than the best combination (Supplemental Table 2).

### Exploratory analysis

Of 125 CNOs, 29 (23%) had full collapse. Both components of the best criteria were associated with CNO severity: Median stenosis EDV was 101 (IQR 45–148) and 150 (IQR 100–213, *p* =.011), and median distal PSV was 18 (IQR 10–42) and 46 (IQR 31–61, *p* <.001) cm/s in CNO with and without full collapse respectively. Among the 121 CNOs with CDU data, with the best criteria, no CNO with full collapse were ruled-out, 12 (46%) ruled-in, 5 (19%) were uncertain and 9 (35%) were mistaken for occlusion. For CNO without full collapse, 2 (2%) were ruled-out, 24 (25%) were ruled-in, 69 (73%) were uncertain and none was mistaken for occlusion.

We applied the best criteria to the non-occluded ICAs that were not used in the main analyses. The results are summarized in Supplemental Fig. 4. In short, < 50% stenoses were almost always (99.5%) ruled-out and the only case ruled-in had unclear cause of small distal ICA. Finally, all 13 ICAs occluded on CTA were also occluded on CDU.

We assessed the CDU variables for conventional stenoses with and without variants, unclear cause of small distal ICA and CNO. All CDU parameters were similar for conventional stenoses with and without variants. For cases with unclear cause of small distal ICA, some CDU parameters were different from conventional stenoses with and without variants, and some were different from CNO (Supplemental Fig. 5).

## Discussion

The main finding of the study was that distal velocity measured by CDU could not separate CNO from conventional ≥ 50% stenosis with sufficient diagnostic accuracy. Hence, criteria to rule-out or rule-in CNO were developed, by which an accurate diagnosis of CNO could be performed in approximately half of the participants. These criteria are intended to be used as part of a diagnostic algorithm, where patients not receiving a certain diagnosis on CDU will require further examinations.

CNO seems to be systematically underdiagnosed [5, 7, 12], and part of this is probably due to difficulties in diagnosing CNO with CDU. This is important as it affects management [6]; If CNO is not identified, patients will undergo unnecessary surgery why a sensitive criterion is needed. Even more important is that conventional stenosis should not be mistaken for CNO and thus being excluded from recommended surgical treatment. Thus, CNO diagnostics must be both sensitive and specific. Previous studies that included only CNO with full collapse showed good sensitivity for low flow velocity in the stenosis [10, 11], but this does not apply for CNO without full collapse. CNO without full collapse are highly relevant as almost all of the CNOs in large trials were without full collapse [1–3, 6]. While distal velocity showed promise in a proof-of-concept study with significant selection issues and overlap with a previous study [12, 13], our larger and systematically gathered data showed that it was not markedly better than other CDU parameters. Also, although a CDU guideline has interpreted low distal velocity as indicative for a hemodynamically significant conventional stenosis [17], low distal velocity is rather indicative of CNO [13]. This notion is further supported by the association between low distal velocity and small distal ICA [18].

It seems reasonable to conclude that CDU is not both sensitive and specific for CNO when including CNO without full collapse: It has now been shown in 3 of 3 materials including a total of 999 participants of which 282 had CNO [5, 12]. Moreover, alternative imaging modalities, such as PC-MRA, have demonstrated markedly higher sensitivity (90%) and specificity (99%) for distinguishing CNO from conventional stenosis of ≥ 50% using a single threshold [8]. This represents a significant advancement compared to earlier threshold-based criteria established for CTA [19]. However, PC-MRA is associated with limitations, including higher costs, limited availability, and contraindications in certain patient populations. Consequently, the ability to diagnose or exclude certain cases of CNO without relying on PC-MRA would be of considerable clinical value in routine practice. Therefore, we present rule-in and rule-out criteria based on the understanding that CDU is conducted as part of a diagnostic algorithm. We chose high sensitivity and specificity cut-offs so that the ruled-in and ruled-out cases would not need further testing. This algorithm should preferably include PC-MRA as a second-line test for unclear cases, but CTA can also be used if it is assessed well [7]. It is important to note that, while this study represents one of the most extensive datasets on CNO diagnostics using CDU, the sample size was insufficient to accommodate both a derivation and a validation cohort. The proposed diagnostic algorithm should be regarded as an initial CDU-based framework designed explicitly for CNO diagnostics, which requires external validation before it can be considered for clinical implementation. Further, while beyond the scope of this study, health economic evaluations are warranted to assess the cost-effectiveness of the proposed novel diagnostic framework for CNO. These evaluations should involve both direct and indirect costs within a cost-per-patient analysis.

Going forward there seems to be three possibilities: Adopt these criteria (or similar future criteria), systematically perform another diagnostic modality than CDU or systematically perform unnecessary surgery on patients with CNO. The latter is an unacceptable policy. The former two both results in that CDU cannot be used as the sole preoperative modality and carotid guidelines that state this should be revised [6, 9]. As our criteria require validation before widespread use, the only currently available alternative is that CDU always is complemented by another diagnostic modality.

### Strengths and limitations

The present study benefits from a large consecutive prospective data collection with state-of-the-art CTA assessments (which is crucial as CNO assessments vary significantly in routine practice [7]). It is unclear if the CTA reference would have been even better had we used standardized CTA protocols. CDU data were collected at a centre where CDU was routinely performed as a clinical investigation by several experienced operators. No systematic evaluation of inter-operator variability or operator training was undertaken for CDU measurements. The possibility that expert CDU laboratories may achieve better results by visualizing more distal segments of the ICA cannot be excluded. Therefore, investigations into the influence of the distance between the stenosis and the distal ICA are warranted. Our data provide no explanation why distal ICA velocity measured with CDU had worse diagnostic performance than distal ICA flow measured with PC-MRA, direct comparisons between these methods are warranted. While CNO is common, 30% of symptomatic ≥ 50% stenosis [4], our even higher CNO prevalence of 40% was likely caused by our selection criteria. Since our screening criteria is based on predefined cut off values for sensitivity and specificity, which are generally considered as stable across different prevalence rates, we believe that higher prevalence is of minor concern regarding our aim to develop a diagnostic CDU algorithm.

## Conclusions

CDU cannot separate CNO and conventional stenosis with sufficient accuracy to be used as a sole preoperative modality, especially not when considering the accuracy of alternative methods, e.g., PC-MRA. With our new screening criteria, CNO could be ruled in or out in approximately half of participants with ≥ 50% stenosis; the other half need further testing, such as with PC-MRA.

**Table 1:** Baseline characteristics

	Conventional ≥ 50% stenosis (*n* = 190)	CNO (*n* = 125)	*P* value
Age, median (IQR), years	75 (71–80)	74 (69–79)	.13^f^
Women, *n* (%)	63 (33)	34 (27)	.32^g^
Symptomatic stenosis, *n* (%)^a^	151 (82)	106 (85)	.54^g^
Current smoker, *n* (%)	31 (16)	22 (18)	.88^g^
Previous stroke, *n* (%)	33 (17)	15 (12)	.21^g^
Coronary artery disease, *n* (%)	51 (27)	29 (23)	.51^g^
Atrial fibrillation, *n* (%)	30 (16)	17 (14)	.63^g^
Diabetes, *n* (%)	61 (32)	30 (24)	.13^g^
Hypertension, *n* (%)	170 (90)	110 (88)	.72^g^
Previous revascularization^b^, *n* (%)	53 (28)	28 (22)	.30^g^
HbA1c, mmol/mol, median (IQR)	42 (29–51)	42 (39–49)	.34^f^
LDL, mmol/L, median (IQR)	1.9 (1.4–2.7)	2.0 (1.6–2.9)	.12^f^
HDL, mmol/L, median (IQR)	1.20 (1.00–1.50)	1.20 (1.00–1.40)	.84^f^
Days between CDU and CTA, median (IQR)	4 (2–7)	3 (1–6)	.18^f^
CTA, Stenosis diameter, mm, median (IQR)	1.4 (1.1–1.7)	0.5 (0.5–0.5)	<.001^f^
CTA, Distal ICA diameter, mm, median (IQR)	4.2 (3.9–4.6)	2.8 (2.3–3.4)	<.001^f^
CTA, ICA ratio^c^, median (IQR)	1.00 (0.97–1.04)	0.67 (0.49–0.76)	<.001^f^
CTA, ECA ratio^d^, median (IQR)	1.64 (1.42–1.89)	1.06 (0.80–1.18)	<.001^f^
CTA, Full collapse, *n* (%)	NA	29 (23%)	NA
CTA, 70–99% stenosis^e^, *n* (%)	71 (37%)	NA	NA

CNO, carotid near-occlusion; HDL, high-density lipoproteins; LDL; low-density lipoproteins; IQR, interquartile range; NA, not applicable

^a^Of 39 asymptomatic participants with conventional stenosis, 32 were asymptomatic bilaterally, 5 had contralateral symptomatic stenosis that were unclear if CNO or conventional and 2 had contralateral symptomatic occlusion. Of 19 asymptomatic participants with CNO, 12 were asymptomatic bilaterally and 7 had contralateral symptomatic stenosis (4 conventional stenosis, 2 unclear if CNO or conventional and 1 < 50%)

^b^Any prior arterial revascularization procedure

^c^Ipsilateral/contralateral distal ICA diameter

^d^Distal ipsilateral ICA/ipsilateral ECA diameter

^e^The degree of conventional stenosis, assessed with NASCET criteria

^f^Mann-Whitney *U* test

^g^Fisher’s exact test

**Table 2:** CDU data of conventional ≥ 50% stenosis and CNO among participants not diagnosed with occlusion on CDU

CDU, median (IQR)	Missing data, *n*/*n*^a^	Conventional ≥ 50% stenosis (*n* = 190)	CNO (*n* = 116)	*P* value
Stenosis PSV, cm/s	0/4	189 (125–272)	395 (289–475)	<.001^e^
Stenosis EDV, cm/s	0/4	56 (39–83)	141 (86–208)	<.001^e^
CCA PSV, cm/s	2/2	66 (54–83)	57 (45–74)	<.001^e^
CCA EDV, cm/s	2/2	15 (12–19)	11 (8–14)	<.001^e^
PSV ratio^b^	2/6	2.8 (1.8–4.5)	6.0 (4.6–8.8)	<.001^e^
EDV ratio^b^	2/7	3.4 (2.5–6.4)	12.6 (7.4–20.2)	<.001^e^
Distal PSV, cm/s	7/3	68 (54–82)	42 (24–60)	<.001^e^
Distal EDV, cm/s	7/3	20 (17–27)	15 (10–21)	<.001^e^
Distal PI^c^	7/3	1.22 (1.02–1.42)	1.09 (0.82–1.25)	<.001^e^
Distal PSV ratio^d^	7/6	0.36 (0.23–0.53)	0.11 (0.07–0.16)	<.001^e^
Distal EDV ratio^d^	7/6	0.39 (0.25–0.58)	0.10 (0.07–0.19)	<.001^e^

CCA, common carotid artery; CDU, colour duplex ultrasound; CNO, carotid near-occlusion; EDV, end-diastolic velocity; IQR, interquartile range; PI, pulsatility index; PSV, peak systolic velocity

^a^Conventional ≥ 50% stenosis/CNO

^b^Stenosis/CCA

^c^PI calculated as (PSV-EDV)/mean velocity, where mean velocity was calculated as EDV+(PSV-EDV)/3

^d^Distal/stenosis

^e^Mann-Whitney *U* test

**Table 3:** Diagnostic performance of single CDU parameters in separating CNO from conventional ≥ 50% stenosis among participants not diagnosed with occlusion on CDU

CDU	AUC (95% CI)	Cutoff^a^	Sensitivity, % (95% CI)	Specificity, % (95% CI)	PPV, % (95% CI)	NPV, % (95% CI)	LR+ (95% CI)	LR- (95% CI)
Stenosis PSV	0.82 (0.77–0.87)	≥ 236	87 (79–92)	69 (62–76)	63 (57–68)	90 (84–93)	2.84 (2.26–3.56)	0.19 (0.12–0.31)
Stenosis EDV	0.81 (0.76–0.86)	≥ 94	73 (64–81)	79 (73–85)	68 (61–74)	83 (79–87)	3.57 (2.64–4.82)	0.34 (0.25–0.46)
CCA PSV	0.62 (0.56–0.69)	≤ 50	38 (29–47)	81 (75–87)	57 (48–66)	67 (63–70)	2.05 (1.41–2.98)	0.76 (0.65–0.89)
CCA EDV	0.73 (0.67–0.79)	≤ 11	59 (49–67)	77 (70–83)	63 (55–69)	74 (69–78)	2.56 (1.89–3.46)	0.54 (0.43–0.67)
PSV ratio^b^	0.82 (0.77–0.87)	≥ 3.8	85 (76–91)	70 (63–76)	62 (56–67)	89 (83–92)	2.72 (2.17–3.41)	0.21 (0.13–0.34)
EDV ratio^b^	0.86 (0.81–0.91)	≥ 5.4	88 (80–93)	71 (64–78)	64 (58–69)	91 (86–95)	3.07 (2.42–3.88)	0.17 (0.10–0.28)
Distal PSV	0.77 (0.71– 0.83)	≤ 49	62 (52–71)	83 (76–88)	69 (61–76)	78 (73–82)	3.54 (2.51–5.01)	0.46 (0.36–0.59)
Distal EDV	0.71 (65–0.77)	≤ 16	58 (48–67)	75 (69–81)	59 (52–66)	74 (70–78)	2.34 (1.73–3.15)	0.56 (0.45–0.71)
Distal PI	0.65 (0.58–0.71)	≤ 1.30	85 (77–91)	36 (29–43)	45 (42–48)	80 (71–86)	1.33 (1.16–1.52)	0.42 (0.26–0.67)
Distal PSV ratio^c^	0.89 (0.86–0.93)	≤ 0.17	77 (68–85)	89 (83–93)	81 (74–87)	87 (82–90)	7.07 (4.62–10.82)	0.26 (0.18–0.36)
Distal EDV ratio^c^	0.87 (0.82–0.92)	≤ 0.25	88 (81–94)	75 (68–81)	68 (62–73)	91 (86–95)	3.51 (2.71–4.55)	0.16 (0.09–0.26)

AUC, area under the curve; CI, confidence interval; CCA, common carotid artery; CDU, colour duplex ultrasound; CNO, carotid near-occlusion; EDV, end-diastolic velocity; LR+, positive likelihood ratio; LR-, negative likelihood ratio; NPV, negative predictive value; PI, pulsatility index; PPV, positive predictive value, PSV, peak systolic velocity

^a^Cutoff values are based on Youden Index

^b^Stenosis/CCA

^c^Distal/stenosis

**Table 4:** Performance of the best single CDU parameter and best combinations of CDU parameters for rule-in and rule-out of CNO with 98% sensitivity and 99% specificity among participants not diagnosed with occlusion on CDU

**Criteria 1**			
**Distal PSV ratio (distal/stenosis) ≥ 0.42**			**Distal PSV ratio (distal/stenosis) ≤ 0.062**
***Rule-out***	***Uncertain***		***Rule-in***
114 participants (39%) 2 CNO NPV^a^: 98% (93–100%) LR-^c^: 0.03 (0.01–0.12)	156 participants (53%) 86 CNO		23 participants (8%) 22 CNO PPV^b^: 96% (75–99%) LR + ^d^: 36 (5.0–267)
**Criteria 2**			
**Stenosis EDV ≤ 65 cm/s and Distal PSV ≥ 28 cm/s**			**Stenosis EDV ≥ 280 cm/s and/or Distal PSV ≤ 23 cm/s**
***Rule-out***		***Uncertain***	***Rule-in***
117 participants (40%) 2 CNO NPV: 98% (94–100%) LR-: 0.03 (0.01–0.11)		141 participants (48%) 74 CNO	35 participants (12%) 34 CNO PPV: 97% (83–100%) LR+: 57 (7.9–407)
**Criteria 3**			
**EDV ratio (stenos/CCA) ≤ 4 and Distal PSV ≥ 29 cm/s**			**EDV ratio (stenosis/CCA) ≥ 30 and/or Distal PSV ≤ 23 cm/s**
***Rule-out***		***Uncertain***	***Rule-in***
109 participants (38%) 2 CNO NPV: 98% (93–100%) LR-: 0.03 (0.01–0.13)		146 participants (51%) 73 CNO	33 participants (11%) 32 CNO PPV: 97% (82–100%) LR+: 54 (7.5–390)
**Criteria 4**			
**Stenosis EDV ≤ 63 cm/s and Distal PSV > 23 cm/s**			**Stenosis EDV ≥ 280 cm/s and/or Distal PSV ≤ 23 cm/s**
***Rule-out***		***Uncertain***	***Rule-in***
115 participants (39%) 2 CNO NPV: 98% (94–100%) LR-: 0.03 (0.01–0.12)		143 participants (49%) 74 CNO	35 participants (12%) 34 CNO PPV: 97% (83–100%) LR+: 54 (7.9–407)

CCA, common carotid artery; CNO, carotid near-occlusion; EDV, end-diastolic velocity; LR+, positive likelihood ratio; LR-, negative likelihood ratio; NPV, negative predictive value; PPV, positive predictive value, PSV, peak systolic velocity

^a^NPV, % (95% CI); ^b^PPV, % (95% CI); ^c^LR-: (95% CI); ^d^LR+: (95% CI)

## Electronic supplementary material

Below is the link to the electronic supplementary material.

Supplementary Material 1
