# Supplementary material for: Rule-out and rule-in of carotid near-occlusion using color duplex ultrasound
Source: Neuroradiology. 2025 Apr 16;67(5):1223–31. doi: 10.1007/s00234-025-03612-2 (PMC12125088; doi:10.1007/s00234-025-03612-2)
Supplement: Supplementary file 1 — Supplementary Material 1 [file 234_2025_3612_MOESM1_ESM.pdf]

SUPPLEMENTAL ONLINE DATA

Rule-out and rule-in of carotid near-occlusion using color duplex ultrasound

Table of Contents

Supplemental Table 1. Diagnostic performance of single CDU parameters in separating CNO from conventional ≥50% stenosis among participants with or without symptoms related to the investigated side of the ICA.

Supplemental Table 2. CDU combinations with <10% more patients classified as uncertain compared to the best algorithm.

Supplemental Figure 1. Three cases of left-sided carotid stenosis on CTA.

Supplemental Figure 2. CDU parameters in patients with conventional ≥50% stenosis and CNOs.

Supplemental Figure 3. Receiver operating characteristic (ROC) curves.

Supplemental Figure 4. Performance of the final CDU algorithm on the non-occluded ICA excluded from the main study analysis.

Supplemental Figure 5. CDU parameters in participants with conventional ≥50% stenosis, conventional ≥50% stenosis with anatomical variant, unclear and CNO.

References

Supplemental Table 1. Diagnostic performance of single CDU parameters in separating CNO from conventional ≥50% stenosis among participants with or without symptoms related to the investigated side of the ICA. Participants diagnosed with occlusion on CDU were excluded.

CDU	Symptomatic AUC (95% CI)	Asymptomatic AUC (95% CI)	*P* value[c]
Stenosis PSV	0.82 (0.77–0.88)	0.77 (0.61–0.93)	.54
Stenosis EDV	0.81 (0.75–0.87)	0.77 (0.62–0.92)	.65
CCA PSV	0.62 (0.54–0.69)	0.64 (0.50–0.78)	.73
CCA EDV	0.73 (0.66–0.79)	0.73 (0.61–0.85)	.96
PSV ratio[a]	0.82 (0.76–0.87)	0.79 (0.65–0.93)	.70
EDV ratio[a]	0.86 (0.81–0.91)	0.84 (0.71–0.97)	.82
Distal PSV	0.79 (0.73–0.85)	0.69 (0.53–0.86)	.30
Distal EDV	0.74 (0.67–0.80)	0.61 (0.44–0.79)	.19
Distal PSV ratio[b]	0.89 (0.84–0.93)	0.91 (0.84–0.98)	.67
Distal EDV ratio[b]	0.87 (0.83–0.92)	0.86 (0.76–0.96)	.82

AUC, area under the curve; CI, confidence interval; CCA, common carotid artery; CDU, colour duplex ultrasound; CNO, carotid near-occlusion; EDV, end-diastolic velocity; PSV, peak systolic velocity.

[a]Stenosis/CCA.

[b]Distal/stenosis.

[c]AUCs were compared according to the method of Hanley and McNeil [1].

Supplemental Table 2. CDU combinations with <10% more patients classified as uncertain compared to the best algorithm. Only participants not diagnosed with occlusion on CDU analysed.

CDU combinations		
**Rule-out**[a]	**Rule-in**[b]	**Uncertain**
Stenosis EDV ≤63 cm/s and<br>Distal EDV ≥11 cm/s	Stenosis EDV ≥280 cm/s and/or<br>Distal EDV ≤8 cm/s	52%
EDV ratio (stenos/CCA) ≤3.8 and<br>Distal EDV ≥8 cm/s	EDV ratio (stenos/CCA) ≥29.5 and/or<br>Distal EDV ≤8 cm/s	54%
EDV ratio (stenos/CCA) ≤3.8 and<br>PSV ratio (distal/stenosis) ≥0.17	EDV ratio (stenos/CCA) ≥29.4 and/or<br>PSV ratio (distal/stenosis) ≤0.05	54%
Stenosis PSV ≤211 cm/s and<br>Distal PSV ≥28 cm/s	Stenosis PSV ≥620 cm/s and/or<br>Distal PSV ≤20 cm/s	54%
Stenosis EDV ≤93 cm/s and<br>PSV ratio (distal/stenosis) ≥0.35	Stenosis EDV ≥280 cm/s and/or<br>PSV ratio (distal/stenosis) ≤0.05	58%

CDU, colour duplex ultrasound; EDV, end-diastolic velocity; PSV, peak systolic velocity.

[a]Predefined as 98% sensitivity

[b]Predefined as 99% specificity

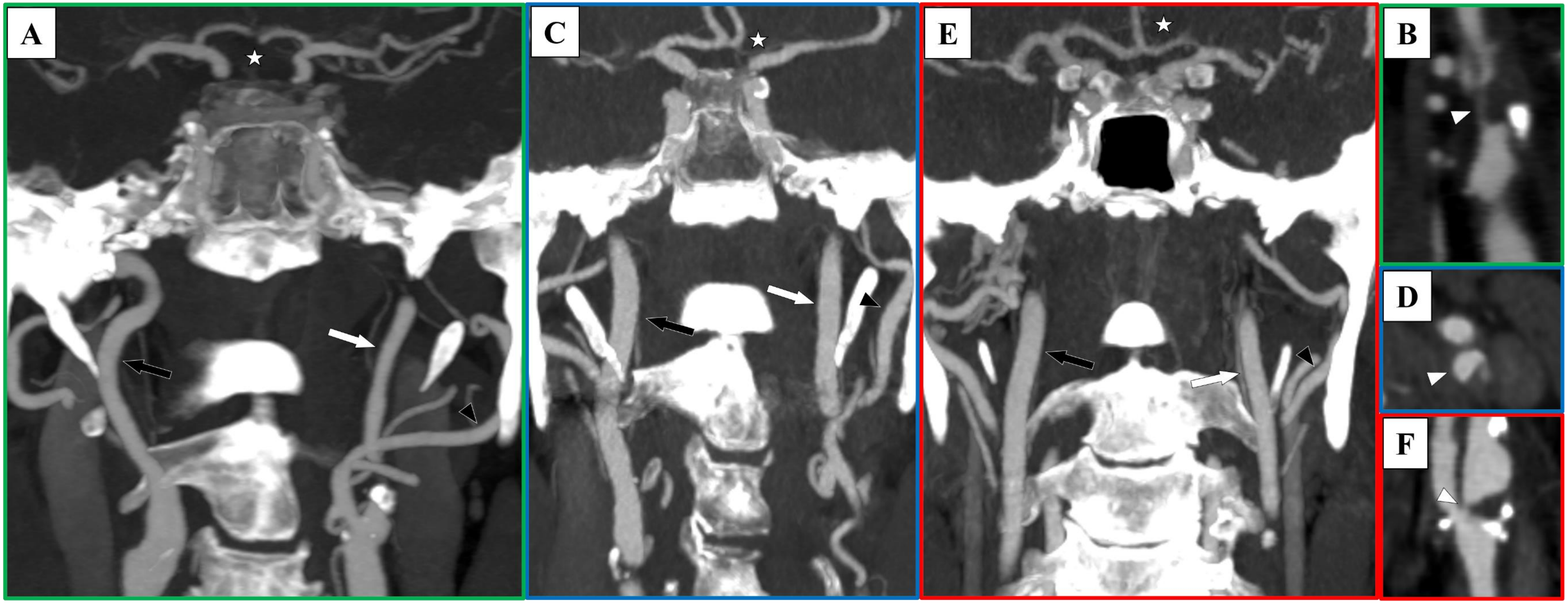

Supplemental Figure 1. Three cases of left-sided carotid stenosis on CTA. **A+B)** CNO without full collapse. After a severe stenosis (assigned as 0.5 mm due to partial volume effect), left distal ICA is small (2.8 mm), smaller than right ICA (3.6 mm) and similar to left external carotid artery (ECA, 2.6 mm). Intracranially, the Circle of Willis is mostly symmetric (A1s looks similar in the illustrated plane). I.e. the smaller ICA is best explained by the stenosis, hence CNO. **C+D)** Conventional stenosis with anatomical variant. After a modest stenosis (2.0 mm), left distal ICA in not very small (4.8 mm, i.e. within normal distribution). While smaller than right ICA (5.8 mm), left ICA is larger than left ECA (3.6 mm). Left A1 is almost not seen. I.e. a hypoplastic A1 explains the ICA asymmetry better than the stenosis, hence anatomical variant. **E+F)** Stenosis with unclear cause of small distal ICA. After a severe stenosis (1.0 mm), left distal ICA is quite small (3.6 mm) and smaller than right ICA (5.0 mm). However, left ICA is still larger than left ECA (2,4 mm) and the left A1 is smaller than right A1. I.e. this could be CNO given severe stenosis and quite small ICA (lower border of normal distribution), but I could also be a variant with moderate A1 hypoplasia given that ICA is larger than ECA. Hence, as the features diverge, the cause of the small distal ICA is unclear. *White arrowhead: Stenosis. White arrow: Distal ICA. Black arrow: Contralateral ICA. Black arrowhead ECA. White star: A1 segments.*

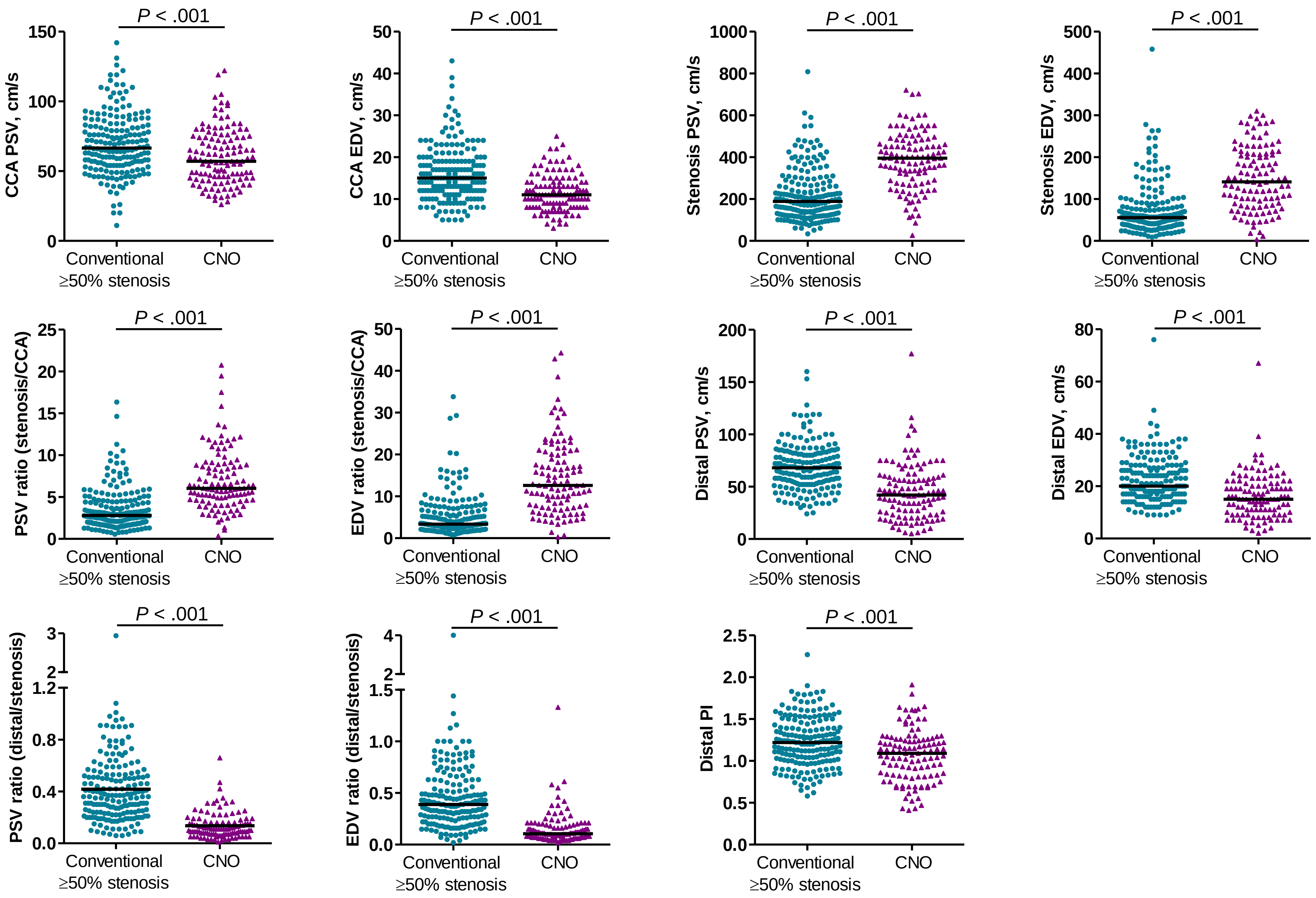

Supplemental Figure 2. CDU parameters in patients with conventional ≥50% stenosis and CNO. Each dot represents one individual and the horizontal bar represent the median. CCA, common carotid artery; CDU, colour duplex ultrasound; CNO, carotid near-occlusion; EDV, end-diastolic velocity; PI, pulsatility index; PSV, peak systolic velocity

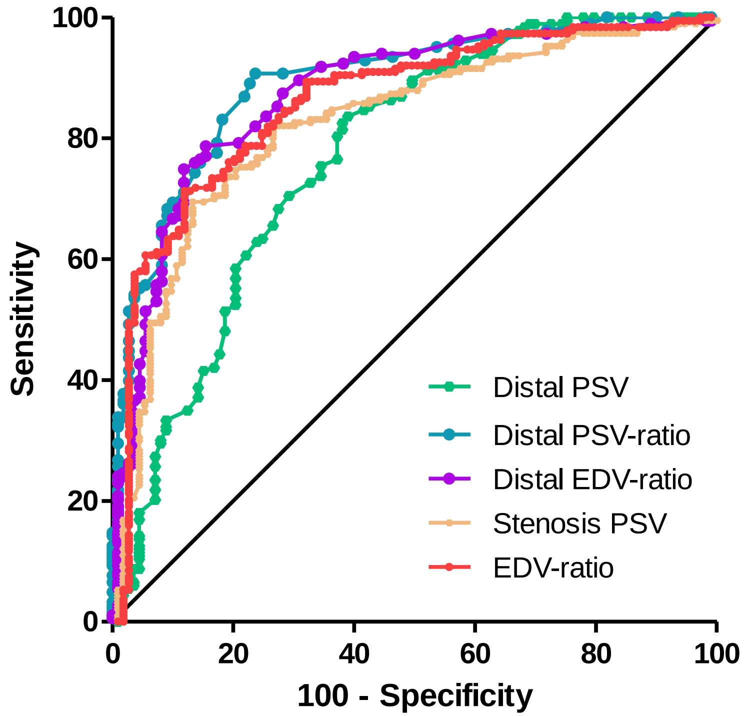

Supplemental Figure 3. Receiver operating characteristic (ROC) curves. The three best parameters to separate near-occlusion and conventional stenosis (distal PSV-ratio, distal EDV-ratio, and EDV-ratio) as well as stenosis PSV and distal PSV. EDV, end-diastolic velocity; PSV, peak systolic velocity; Distal PSV-ratio, distal/stenosis; EDV-ratio, stenosis/CCA.

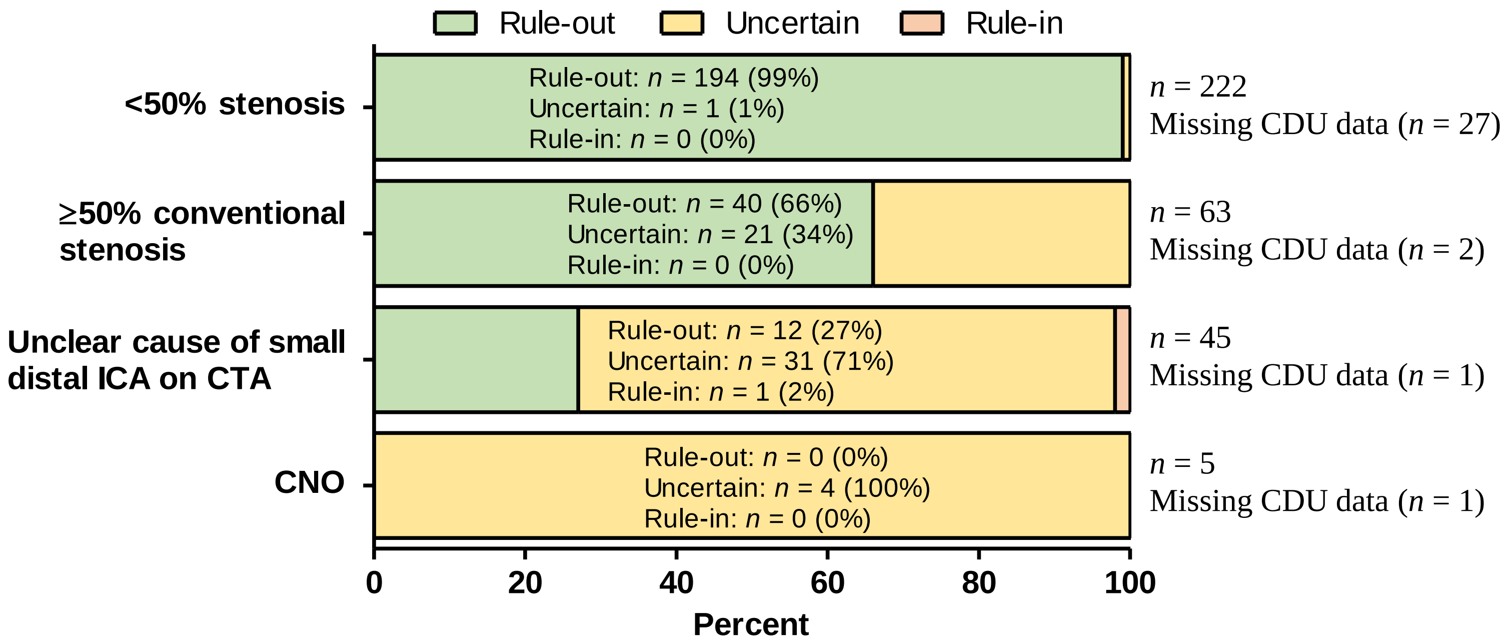

Supplemental Figure 4. Performance of the final CDU algorithm on the non-occluded ICA excluded from the main study analysis.· Analyzed conventional ≥50% stenosis and CNOs are contralateral to the stenosis examined in the main study. CDU, colour duplex ultrasound; CNO, carotid near-occlusion.

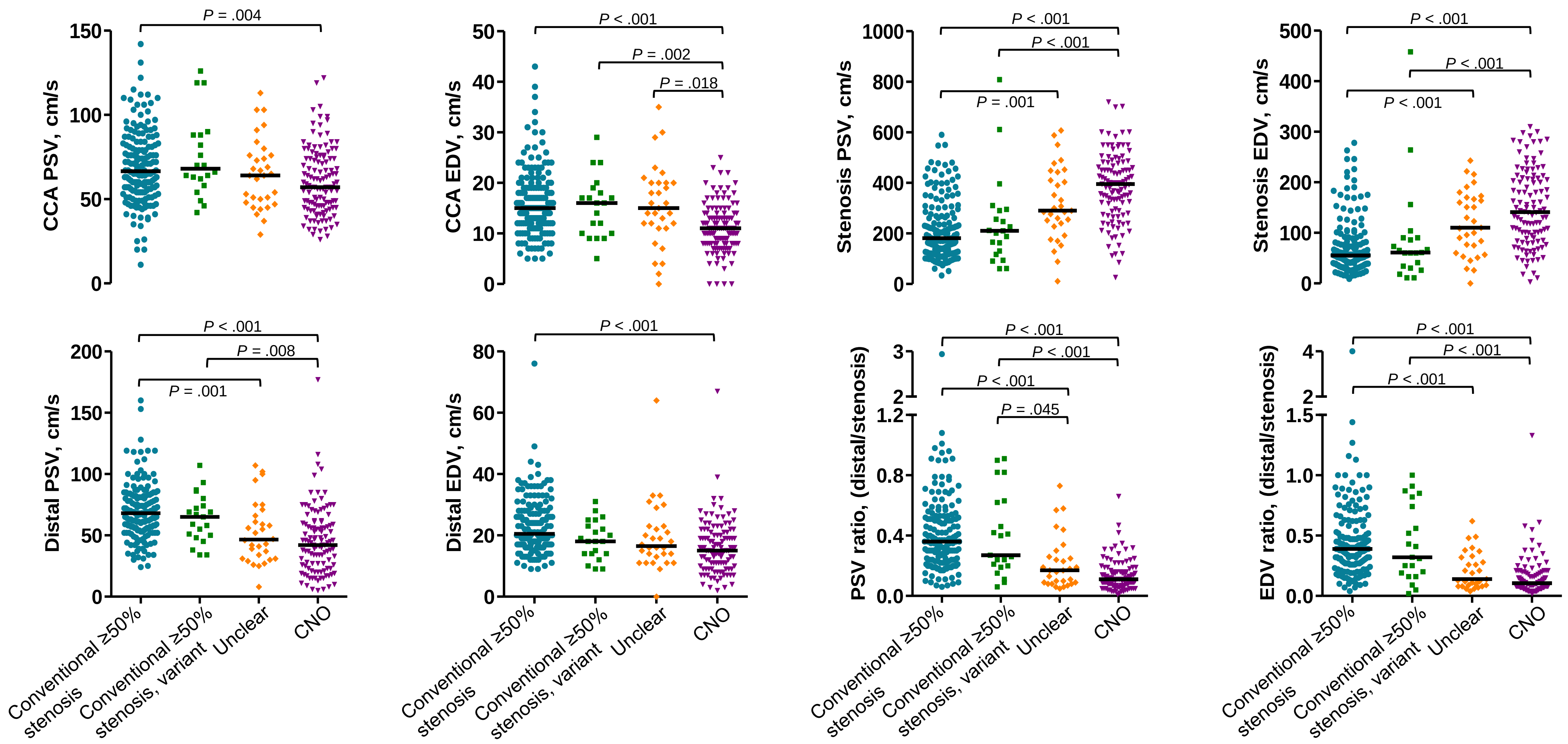

Supplemental Figure 5. CDU parameters in participants with conventional ≥50% stenosis, conventional ≥50% stenosis with anatomical variant, unclear and CNO. The "unclear" cases are those with unclear cause of small distal ICA on CTA. Each dot represents one individual, and the horizontal bar represent the median. CCA, common carotid artery; CDU, colour duplex ultrasound; CNO, carotid near-occlusion; EDV, end-diastolic velocity; PSV, peak systolic velocity. Between-group differences were examined using the Kruskal-Wallis test, followed by Dunn-Bonferroni corrected post hoc pairwise comparisons.

References

1. Hanley JA, McNeil BJ. The meaning and use of the area under a receiver operating characteristic (ROC) curve. Radiology. 1982;143(1):29-36. doi: 10.1148/radiology.143.1.7063747.
